# Automated Assessment of *β*-Cell Area and Density per Islet and Patient Using TMEM27 and BACE2 Immunofluorescence Staining in Human Pancreatic *β*-Cells

**DOI:** 10.1371/journal.pone.0098932

**Published:** 2014-06-06

**Authors:** Markus P. Rechsteiner, Xenofon Floros, Bernhard O. Boehm, Lorella Marselli, Piero Marchetti, Markus Stoffel, Holger Moch, Giatgen A. Spinas

**Affiliations:** 1 Institute of Surgical Pathology, University Hospital Zurich, Zurich, Switzerland; 2 Department of Computer Science, ETH Zurich, Zurich, Switzerland; 3 Division of Endocrinology, Diabetes and Metabolism, Ulm University Medical Centre, Ulm, Germany; 4 Department of Clinical and Experimental Medicine – Islet Cell Laboratory, University of Pisa, Pisa, Italy; 5 Institute of Molecular Systems Biology, ETH Zurich, Zurich, Switzerland; 6 Division of Endocrinology, Diabetes and Clinical Nutrition, University Hospital Zurich, Zurich, Switzerland; University of Michigan Medical School, United States of America

## Abstract

In this study we aimed to establish an unbiased automatic quantification pipeline to assess islet specific features such as *β*-cell area and density per islet based on immunofluorescence stainings. To determine these parameters, the *in vivo* protein expression levels of TMEM27 and BACE2 in pancreatic islets of 32 patients with type 2 diabetes (T2D) and in 28 non-diabetic individuals (ND) were used as input for the automated pipeline. The output of the automated pipeline was first compared to a previously developed manual area scoring system which takes into account the intensity of the staining as well as the percentage of cells which are stained within an islet. The median TMEM27 and BACE2 area scores of all islets investigated per patient correlated significantly with the manual scoring and with the median area score of insulin. Furthermore, the median area scores of TMEM27, BACE2 and insulin calculated from all T2D were significantly lower compared to the one of all ND. TMEM27, BACE2, and insulin area scores correlated as well in each individual tissue specimen. Moreover, islet size determined by costaining of glucagon and either TMEM27 or BACE2 and *β*-cell density based either on TMEM27 or BACE2 positive cells correlated significantly. Finally, the TMEM27 area score showed a positive correlation with BMI in ND and an inverse pattern in T2D. In summary, automated quantification outperforms manual scoring by reducing time and individual bias. The simultaneous changes of TMEM27, BACE2, and insulin in the majority of the *β*–cells suggest that these proteins reflect the total number of functional insulin producing *β*–cells. Additionally, *β*–cell subpopulations may be identified which are positive for TMEM27, BACE2 or insulin only. Thus, the cumulative assessment of all three markers may provide further information about the real *β*–cell number per islet.

## Introduction

Assessment of *β*-cell area and density resulting in actual *β*-cell number per islet in human type 2 diabetes is notoriously difficult and a precise calculation of *β*-cell mass is only possible if the weight of the pancreas is known. We have previously shown that the transmembrane protein 27 (TMEM27) and the *β*-site amyloid precursor protein cleaving enzyme (BACE2) are coexpressed in pancreatic *β*-cells in mice [1]. Furthermore, pancreatic islets of mice lacking functional BACE2 have elevated TMEM27 expression that correlates with an increase in *β*-cell mass and improved glucose homeostasis. These results corroborate earlier findings that revealed a growth promoting and insulin stimulatory activity of TMEM27 in pancreatic *β*-cells [2], [3]. Recently, *β*-cell mass was successfully monitored using fluorescence and radioactively labeled anti-TMEM27 antibody in mice [4]. Esterhazy et al. also provided preliminary evidence that TMEM27 and BACE2 are coexpressed in human islets and that pancreatic tissue sections of patients with T2D exhibited decreased *β*-cell area per islet as compared with ND [1]. In that dataset, TMEM27 and BACE2 expression were heterogenous among islets within the same individual rendering manual quantification difficult and prone to sampling errors. Recently, a novel detection algorithm for scoring immunohistochemically stained sections and extracting islet-specific features was developed [5]. This algorithm is based on automatically detecting the nuclei in a tissue section and defining a cell to be positively stained depending on its stained surrounding or nuclear staining. However, the analysis of an immunohistochemically stained section allows only the extraction of one stained layer. Thus, double stainings in immunohistochemistry which represent proteins in the same cell and same intracellular location are difficult to interpret. To overcome this issue we adapted the algorithm developed for scoring immunohistochemically stained sections for the detection of immunofluorescence stainings [1], [5], [6].

In the present study, we applied and validated this automated approach in a large cohort of pancreatic tissue specimens. By integration of staining intensity and stained area per islet, we were able to determine the *β*-cell area which is termed hereafter as ‘area score’. The TMEM27 and BACE2 area scores were significantly lower in T2D as compared ND. Furthermore, TMEM27, BACE2 and insulin area scores correlated significantly. Additionally, islet size and *β*-cell density per islet based either on TMEM27 or BACE2 staining correlated significantly.

## Materials and Methods

### Study design and patients

The dataset comprised tissue specimens of 32 patients with T2D and of 28 ND, including those reported previously [1]. The tissue specimens (autopsy, surgery, cadaveric donors) were collected by the Institute of Surgical Pathology, University Hospital Zurich, the Department of Clinical and Experimental Medicine – Islet Cell Laboratory, University of Pisa and the Division of Endocrinology, Diabetes and Metabolism, Ulm University Medical Centre. Clinical data are summarized in Table 1. The presence of type 2 diabetes was diagnosed if glycosylated haemoglobin A1c [HbA1c] was higher than 7%, or fasting plasma glucose [FPG] higher than 6.9 mmol/l and/or the diagnosis confirmed in the medical record. Exclusion criteria were specific forms of diabetes, autolytic pancreatic tissue, evidence of pancreatitis, presence of lymphomas or insulinomas or treatment with immunosuppressive drugs.

**Table 1:** Clinical characteristics of study subjects.

Gender (M/F)	Age (years)	BMI (kg/m∧2)	Antidiabetic treatment	Source	*Location	Number of islets pictured		
Non-diabetic						TMEM27	BACE2	Insulin
M	51	21	no	Surgery	head	10	10	20
M	56	32	no	Surgery	head	10	3	13
F	30	28	no	Surgery	tail	7	10	20
M	54	25	no	Surgery	head	10	10	20
F	46	31	no	Surgery	tail	10	10	20
F	49	24	no	Surgery	head	10	10	20
M	78	29	no	Surgery	head	8	10	12
M	72	21	no	Autopsy	body	9	10	20
F	89	25	no	Autopsy	body	10	10	20
F	81	25	no	Autopsy	body	11	10	20
F	74	24	no	Autopsy	body	10	8	20
M	63	28	no	Cadaveric donor	body	9	10	20
M	78	24	no	Cadaveric donor	body	8	10	14
M	76	26	no	Cadaveric donor	body	10	10	20
F	70	29	no	Cadaveric donor	body	5	3	4
F	62	26	no	Cadaveric donor	body	5	10	15
F	78	26	no	Cadaveric donor	body	4	10	10
M	82	28	no	Surgery	head	10	10	9
F	76	28	no	Surgery	head	9	10	17
F	55	26	no	Surgery	body	4	10	15
M	71	24	no	Surgery	head	10	10	10
M	72	29	no	Surgery	head	10	10	10
F	57	19	no	Surgery	body	10	10	10
F	50	23	no	Surgery	head	10	10	10
F	77	28	no	Surgery	body	7	10	20
F	61	26	no	Surgery	body	3	10	18
F	69	29	no	Surgery	body	6	10	15
M	57	24	no	Surgery	body	7	5	20
Mean	66	26						
SDM	14	3						
**Type 2 diabetes**								
F	66	29	Insulin	Surgery	head	10	5	20
F	62	32	Metformin	Surgery	body	12	2	13
M	70	35	Diet	Surgery	head	9	5	20
M	63	31	Metformin	Surgery	head	9	5	20
M	68	33	Metformin	Surgery	tail	6	15	20
F	83	23	Diet	Autopsy	body	10	5	20
M	77	26	Metformin	Autopsy	body	10	10	15
F	74	23	Metformin	Autopsy	body	10	10	12
M	61	28	Insulin	Cadaveric donor	body	11	10	20
M	66	23	Metformin	Cadaveric donor	body	10	10	20
F	53	30	Metformin	Cadaveric donor	body	1	9	20
F	54	24	Metformin	Cadaveric donor	body	10	10	20
F	75	27	Metformin	Cadaveric donor	body	6	10	14
F	62	30	Insulin	Surgery	not specified	10	10	20
F	78	34	Diet	Surgery	not specified	6	6	4
M	79	36	Diet	Surgery	head	6	1	15
M	60	32	Insulin	Surgery	head	10	10	10
M	45	34	Diet	Autopsy	head	10	10	12
F	59	33	Metformin	Autopsy	body	10	10	12
F	74	31	Sulfonylurea	Autopsy	head	10	10	12
M	59	32	Diet	Autopsy	body	10	10	12
M	80	29	Metformin	Autopsy	head	10	10	12
F	54	30	Metformin	Autopsy	head	10	10	12
F	63	32	N/A	Autopsy	head	10	10	12
M	58	31	Metformin	Autopsy	body	10	10	12
M	53	34	Metformin	Autopsy	body	10	10	12
F	55	33	Metformin	Autopsy	body	10	10	12
M	51	36	N/A	Autopsy	head	10	10	12
M	68	28	Sulfonylurea	Autopsy	head	10	9	12
F	54	29	Metformin	Autopsy	body	10	10	12
M	56	31	Metformin	Autopsy	head	10	10	12
M	57	34	Sulfonylurea	Autopsy	body	10	10	12
Mean	64	30						
SDM	10	4						

*Pancreatic tissue was obtained from cadaveric organ donors (6 non-diabetic patients/6 type 2 diabetic patients), autopsies (4 non-diabetic patients/18 type 2 diabetic patients) or from surgically resected pancreatic tissue due to carcinoma of the pancreas of pancreatic duct (19 non-diabetic patients/9 type 2 diabetic patients).

### Ethics Statement

The study was approved by the Cantonal Ethics Committee of Zurich and waived the need for consent (KEK-ZH-NR: StV 29-2006).

### Immunofluorescence

**Figure 1:**
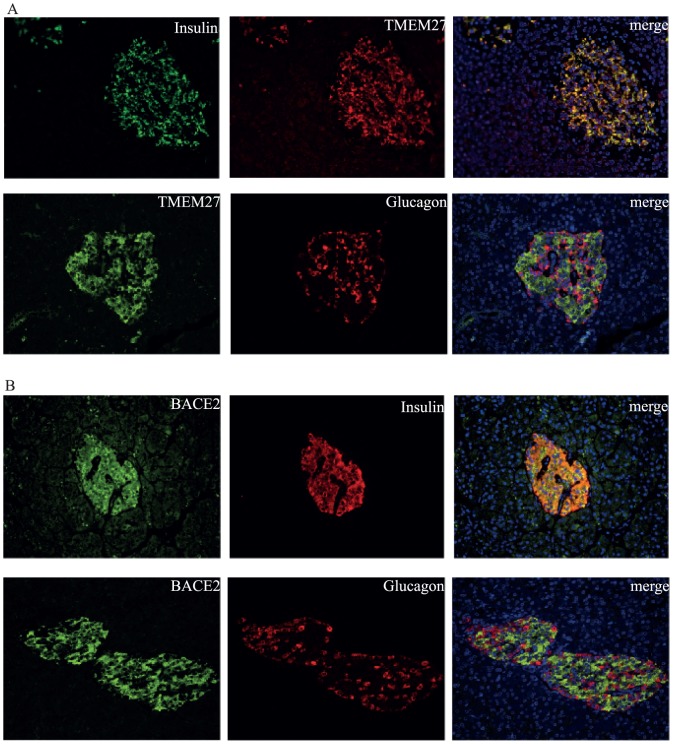
Expression of TMEM27 and BACE2 in pancreatic human islets. Colocalization of insulin and TMEM27 was found in pancreatic *β*-cells, whereas no TMEM27 was detected in glucagon positive *α*-cells (**A**). BACE2 and insulin are coexpressed in pancreatic *β*-cells (**B**).

Antibodies and procedures were the same as described [1]. Primary antibodies used were rabbit anti-mouse TMEM27/Collectrin4 (used in Figure 1A, upper panel), mouse anti-human TMEM27 9/28 (Roche, Switzerland; used in Figure 1A, lower panel), mouse anti-human BACE2 1/9 (Roche, Switzerland), guinea pig anti-human insulin (Linco Research, USA), and rabbit anti-human glucagon (Novocastra Laboratories Ltd, UK). Fluorescence pictures were taken with a resolution of 1376×1032×3 pixels and 20× magnification. Raw unedited material was used for the automated analysis pipeline. The stainings were done on serial cuts of the same tissue block.

### Automated immunofluorescence scoring and islet-specific feature extraction

In this work, the main hypotheses are validated by both a manual immunofluorescence scoring by a pathologist and an automated unbiased staining estimator. The automated immunofluorescence scoring attempts to approximate and mimic the approach followed by a pathologist as described in Esterhazy et al [1], where the scoring was assessed manually without knowledge of the clinical diagnosis.

The manual scoring is estimated by a pathologist who takes into account two factors: the intensity of the staining, as well as the percentage of cells which are stained within an islet. Those two factors are combined in an appropriate way to give a manual estimator in the range [0,3], with an accuracy of 0.25. The decision tree which describes this process, is depicted in Figure S1A. To compensate for “subjective” scoring bias, all images are presented randomly to the pathologist and the whole procedure is repeated three times, where in each time the images are shuffled. Finally, the mean score, over the three repetitions, per islet and specimen is reported. Representative pictures for the scoring scheme are shown in Figure S1B.

The automated immunofluorescence scoring approach, proposed in this work, aims at providing an unbiased and objective staining estimate, which reflects the intuition of the pathologist's approach and can be automatically and efficiently computed. The implemented pipeline which provides the automated estimator, as well as other islet-specific features, is depicted in Figure S2 and consists of the following steps: (a) At first, for each image, the pathologist provides a segmentation of the contained islet. This segmentation could be also performed automatically following the framework in [6]. However, this would introduce extra segmentation bias and error, which could potentially affect the hypotheses testing of the current work. (b) Then, based on the dapi channel information, the cell nuclei are detected using the approach in [5] as implemented in the TMarker software [http://comp-path.inf.ethz.ch/]. (c) A crucial step follows where the parts of the image considered as stained are separated from the background. To achieve that, a k-means algorithm, with k = 2, is employed to cluster the intensity histogram into two groups, namely staining and background. This approach has the advantage that it does not require any threshold to be set for every image, while being able to adaptively remove the background signal and be invariant to illumination artefacts. (d) Knowing the histogram distribution of the staining and background signal allows us to classify each pixel into the respective classes of either being positively stained or belonging into the background. (e) To classify as *α-* or *β*-cells, we look at a patch around each nuclei and count the total number of stained pixels in the patch for both the 555 and 488 channels. Using a majority voting scheme on those pixel sums, we can evaluate if there exists more evidence that the cell can be classified as *α-*cells (555 channel) or *β*-cells (488 channel). (f) The final automated estimator can be now computed as the total number of pixels that are classified as stained in the islet (excluding the nuclei areas) normalized with the area of the islet. Because the automated estimator needs to be comparable with the manual one, it is linearly rescaled into the [0,3] interval. Other islet-specific features can be also evaluated. For example, the *β*-cell density per islet is computed as the number of cells classified as positively stained over background and normalized to the islet area. We included the MatLab code as Materials and Methods S1.

### Immunohistochemistry

For immunohistochemical staining we used the Ventana Benchmark platform with a standard antigen retrieval program (pressure cooking). Mouse anti-human insulin (Novocastra Laboratories Ltd, UK) was used in a dilution of 1∶80 and UView DAB anti-polyvalent as secondary antibody for detection. All islets were manually defined by their morphology. Pictures were taken with a resolution of 1376×1032×3 pixels and 20× magnification. Whole islet area and percent insulin positive area per islet were calculated using the software analySIS (Olympus Biosystems GmbH). The percent insulin positive area was then linearly rescaled into a [0,3] interval to match the automated and manual scoring area values. The stainings were done on serial cuts of the same tissue block as used for immunofluorescence stainings.

### Islet selection and picturing

Only islets which showed intact morphology and accurate staining were analysed. The exact number of islets per patient are summarized in Table 1. In average, nine islets were pictured for TMEM (3–12), nine for BACE (1–15), and 15 for insulin (4–20). In total 1974 (TMEM27 n = 528, BACE2 n = 541, insulin = 905) islets were pictured. For all pictures, scoring was done manually with randomly shuffled pictures and automatically. Islet size was determined three times independently with randomly shuffled pictures.

### Statistical analysis

Differences between means were assessed by unpaired, two-tailed Student's t-test with a confidence interval of 95%. Pearson's correlation coefficient r^2^ and according *p-values* were calculated two-tailed and with a confidence interval of 95%. Analysis of covariance (ANCOVA) was applied to fit linear models. *p*<0.05 was considered significant.

## Results

### Automated extraction of islet specific features

To assess TMEM27 and BACE2 expression in *β*-cells tissue sections were stained with the specific antibody together with insulin or glucagon staining. As depicted in Figures 1A and B, TMEM27 and BACE2 specifically costained with insulin as a marker for *β*-cells whereas no costaining was found with glucagon as a marker for *α*-cells.

In total 1069 (TMEM27 n = 528, BACE2 n = 541) islets stained with either mouse anti-human TMEM27 9/28 or mouse anti-human BACE2 1/9 together with rabbit anti-human glucagon were selected, pictured, and scored for the *β*-cell positive area per islet using the scoring system described in Figure S1. Manual scoring of the immunofluorescence pictures of islets stained for TMEM27 and BACE2 showed a high heterogeneity among islets within the same individual which made quantification of expression difficult.

To provide unbiased quantitation and to increase sensitivity, the pictures were separated into three layers representative for the costainings (target 1, target 2, nucleus) and processed using the automated quantification pipeline. The resulting TMEM27 and BACE2 area scores were highly variable and comparable to what was found by manual scoring (Figure S3A-B). Costaining of TMEM27 or BACE2 together with glucagon allowed us to determine the islet size which was also very heterogenous (Figure S3C-D). Furthermore, cells defined as positive by TMEM27 or BACE2 staining together with the known size of the islets resulted in the *β*-cell density per islet (Figure S3E-F). The degree of variation was similar for all parameters both in type 2 diabetic patients and non-diabetic individuals, respectively.

No differences were observed in TMEM27 or BACE2 staining when comparing ND or T2D grouped into autopsy/cadaveric organ donor vs. surgery. Moreover, stainings did not differ between different ischemic times, which were available for the cadaveric donor group. Neither TMEM27 nor BACE2 showed a different staining pattern or expression level depending on the location where tissue specimens were taken (pancreatic tail vs. body vs. head).

### Correlation of *β*-cell area scores, islet size, and *β*-cell density

**Figure 2:**
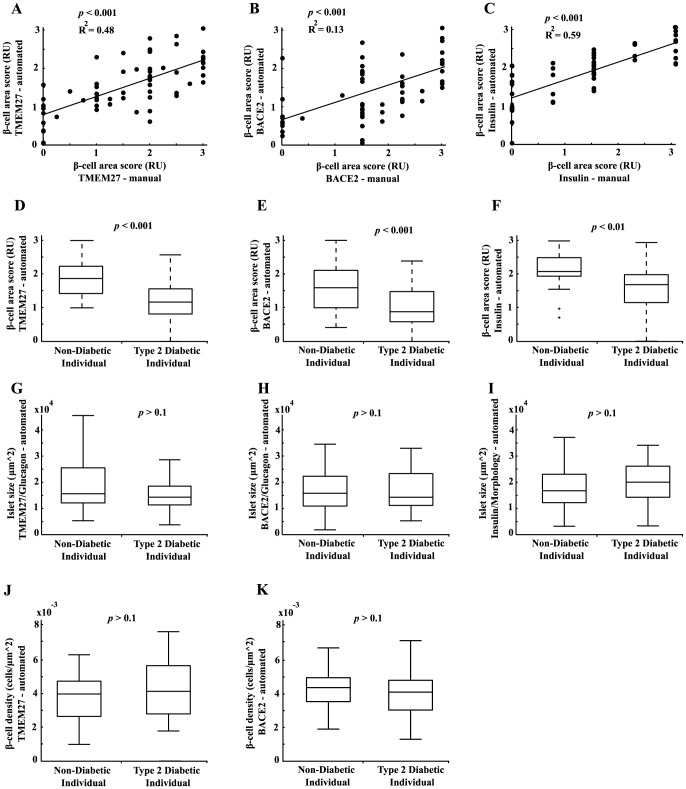
Assessment of *β*-cell area scores, islet size, and *β*-cell density. Correlation between the median of manually obtained area scores and the median of automated area scores per patient in the whole cohort (non-diabetic patients and type 2 diabetic patients) for TMEM27 (**A**), BACE2 (**B**), and insulin (**C**). Median area scores of all non-diabetic patients vs. all type 2 diabetic patients for TMEM27 (**D**), BACE2 (**E**), and insulin (**F**). Median islet size of all non-diabetic patients vs. all type 2 diabetic patients assessed either by TMEM27/glucagon (**G**), BACE2/glucagon (**H**), or insulin/morphology (**I**). Median *β*-cell density of all non-diabetic patients vs. all type 2 diabetic patients assessed either by TMEM27 (**J**) or BACE2 positive cells (**K**). Values in Figure D-K were assessed by the automated pipeline.

In addition to the immunofluorescence stainings for TMEM27 and BACE2, insulin expression was assessed by immunohistochemistry (IHC) which is nowadays used as gold standard in pathology to determine the amount of *β*-cells in human pancreatic islets. The insulin positive area per islet of 905 islets in total was determined automatically using the commercial software analySIS from Olympus and manually using the scoring system described in Figure S1. The resulting median area scores of all islets per patient are plotted in Figure 2. We found a highly significant correlation between automatically and manually scored specimens with regard to TMEM27 (R^2^ = 0.48, *p*<0.001), BACE2 (R^2^ = 0.13, *p*<0.001), and insulin (R^2^ = 0.59, *p*<0.001), respectively (Figure 2A-C).

The median of the automatically assessed area scores of all T2D revealed significant lower TMEM27 (*p*<0.001), BACE2 (*p*<0.001), and insulin (*p*<0.01) values as compared to pancreatic tissue of ND (Figure 2D-F). Combining the three area scores and taking the mean per patient resulted in the most stringent separation of ND and T2D (data not shown). Of note, manually assessed TMEM27 and insulin area scores were also significantly different between ND and T2D. A lower, albeit not significant, islet size was observed in T2D as assessed by costaining of TMEM27/glucagon or BACE2/glucagon and in the *β*-cell density assessed by BACE2 staining (Figure 2 G-K).

**Figure 3:**
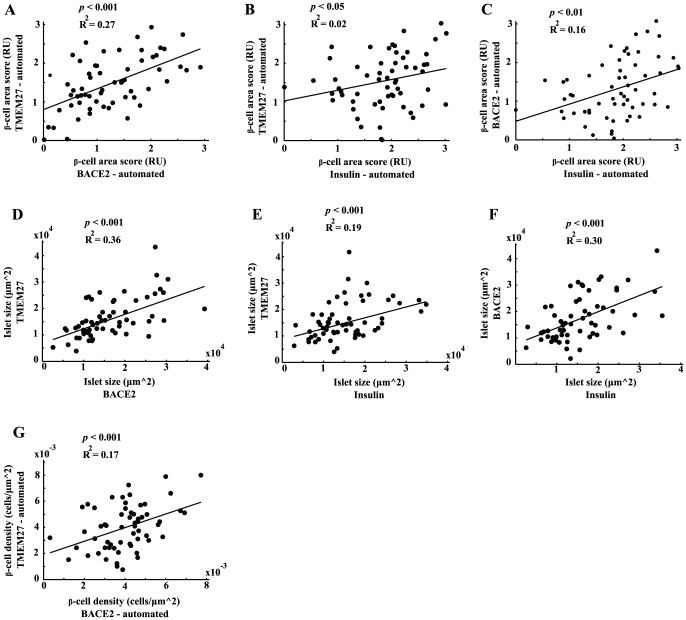
Extraction of islet specific features per individual patient. Correlation of TMEM27 and BACE2 (**A**), TMEM27 and insulin (**B**), and BACE2 and insulin area scores per patient (**C**). Correlation of islet size assessed either by TMEM27, BACE2, and insulin per patient (**D-F**). Correlation of *β*-cell density assessed either by TMEM27 or BACE2 positive cells per patient (**G**). Values in Figure A-G were assessed by the automated pipeline and points represent the median values per parameter and patient.

The median of automatically assessed TMEM27 and BACE2 area scores correlated significantly in each individual patient (R^2^ = 0.27, *p*<0.001, Figure 3A). The insulin area score, which was assessed by IHC, correlated as well with TMEM27 (R^2^ = 0.02, *p*<0.05) and BACE2 (R^2^ = 0.16, *p*<0.01) as depicted in Figures 3B-C. Costaining of *β-*cells and *α-*cells using immunofluorescence revealed a significant correlation between islet size defined either by TMEM27 (*β*-cells) and glucagon (*α-*cells) staining or by BACE2 (*β*-cells) and glucagon staining. Moreover, islet size defined immunohistochemically by insulin staining and by the morphological separation of endocrine and exocrine tissue correlated as well with the islet sizes described above (R^2^ = 0.36, *p*<0.001; R^2^ = 0.19, *p*<0.001; R^2^ = 0.3, *p*<0.001; Figure 3D-F). Furthermore, *β*-cell density based on either TMEM27 or BACE2 positive cells correlated significantly (R^2^ = 0.17, *p*<0.001; Figure 3G).

### Correlation of extracted parameters with body mass index

TMEM27 and BACE2 expression might be involved in regulating the *β*-cell number not only in a disease state like type 2 diabetes but also in other conditions associated with increased insulin demand such as the metabolic syndrome or obesity. We therefore related the area scores of TMEM27, BACE2, and insulin to the BMI.

**Figure 4:**
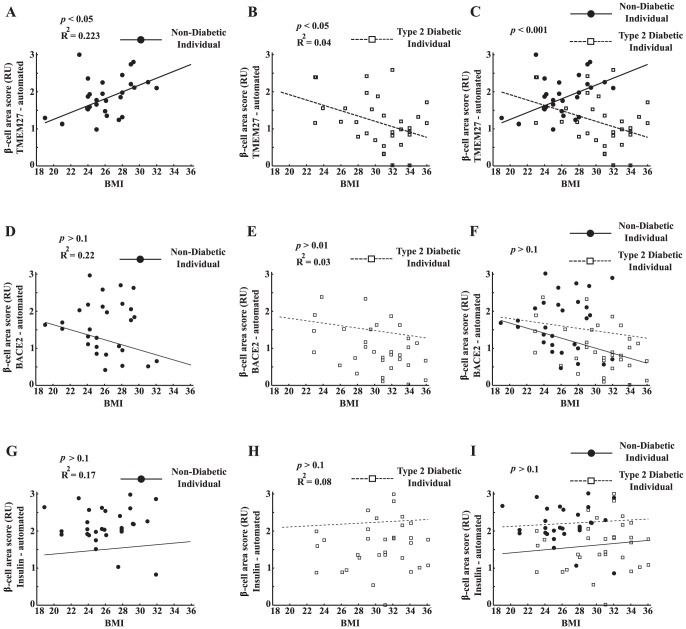
Correlation of TMEM27, BACE2, and insulin area scores to BMI. Automated area scoring of TMEM27 is presented as median of individual patients and correlated to BMI of non-diabetics (straight line; **A**) and patients with type 2 diabetes (dotted line; **B**). The slopes of the fitted lines were significantly different for the TMEM27 area scores (**C**). The same is plotted for BACE2 (D-F) and insulin (H-J).

We found a significant positive correlation between TMEM27 area score and body mass index (BMI) in the ND (R^2^ = 0.223, *p*<0.05; Figure 4A) and a negative correlation in T2D (R^2^ = 0.04, *p*<0.05; Figure 4B). After correlating TMEM27 to BMI of ND and T2D separately, the resulting slopes were significantly different (*p*<0.001, Figure 4C). BACE2 and insulin expression did not correlate with BMI (Figure 4 D-J). By combining the three values of the area scores and taking the mean per patient did not result in a more stringent separation of the two slopes (ND vs. T2D) than considering TMEM27 area scores alone (data not shown).

## Discussion

In the present study using a large and unique dataset of 60 pancreas biopsies, cadaveric organ donors, and autopsy material of T2D patients and ND individuals, we demonstrate that an automated quantification approach of histopathological sections stained by immunofluorescence yields reliable and consistent results, thus saving time of analysis and diminishing subjective error rates.

Several approaches are currently tested for direct imaging of *β*-cells in humans. One of the most promising approaches is the positron emission tomography (PET). However, PET is very challenging due to the heterogenous distribution and the small number of *β*-cells throughout the pancreas [7]. Another highly promising method was presented by Brom and co-workers who traced *β*-cells in humans by SPECT and could demonstrate, at least in a rat model, that the results correlated with *β*-cell mass [8]. Thus, imaging *in vivo* and determination of *β*-cells in humans remains still an unresoved issue. Morphometric analysis on biopsies represents another way to determine the approximate number of *β*-cells. Currently, medical imaging of biopsies for diagnosis relies mostly on immunohistochemistry (IHC). However, IHC stainings are hard to quantitate and allow only to extract one stained layer, making the interpretation of costainings difficult. To resolve this methodological problem and to improve quantification of costainings in pancreatic islets, we adapted a recently developed automated computational pathology scoring system described previously [5], [6]. Contrary to other approaches, we do not directly employ the intensity values of the image pixels in the final staining estimator resulting in *β*-cell area scores and *β*-cell density. Based on internal tests we found this approach to be more robust to staining artifacts and staining variability across the images. However, it should be noted that the intensity of the staining is taken indirectly into account in the first step where we classify the pixels as stained or not based on their intensity exceeding the background estimation.

Using our adapted detection algorithm, we were able to validate our previous findings [1], demonstrating that TMEM27 and BACE2 are *β*-cell specific and costain with insulin. Manual and automated *β*-cell area scoring correlated significantly for TMEM27, BACE2, and insulin which was used as gold standard in this study. Moreover, TMEM27, BACE2, and insulin *β*-cell area scores were similar in each individual patient. These findings underscore the specificity of our automated detection system as the expression levels of TMEM27 and BACE2 were assessed by immunofluorescence and insulin by immunohistochemistry. Additionally, two different analysing algorithms were used to generate these results. This highlights the method as a reliable high-throughput analysis tool for possible routine applications in diagnostic procedures. It should, however, be noted that in some patients substantial differences between the area scores of TMEM27, BACE2, and insulin were observed. We therefore hypothesize that specific *β*-cell subpopulations exist, which express one or the other marker to a different degree. Such a scenario was indeed proposed by Talchai and co-workers, who identified de-differentiated *β*-cells under stress conditions, which lost their expression of insulin [9], [10]. Whether such insulin negative de-differentiated *β*-cells still express TMEM27 or BACE2 (and if the process is reversible) remains elusive. If so, TMEM27 and BACE2 may represent valuable markers to track the real number of potentially functional *β*-cells.

The median *β*-cell area scores of TMEM27, BACE2 and insulin were significantly decreased in T2D as compared to ND. This finding is in line with previous publications, where TMEM27 expression in RNA extracts of whole islets was found to be decreased in diabetic patients compared to non-diabetic individuals [11], [12]. Whereas we found islets with no detectable TMEM27 expression, BACE2 was expressed in every islet above background staining. The ubiquitous presence of BACE2 in human islets points to an important role of BACE2 in regulating TMEM27 and *β*-cell mass [1]. As opposed to *β*-cell area scores median islet size and *β*-cell density did not differ significantly between T2D and ND. It is however important to note that all three extracted islet specific features (*β*-cell area score, islet size, and *β*-cell density) either derived from TMEM27, BACE2 or insulin positive cells correlated significantly in each individual patient, which underscores again the robustness of our approach.

An intriguing observation of the present study was that the TMEM27 area score in islets correlated positively with BMI in non-diabetic individuals. This finding could be viewed as reflecting the compensatory increase in *β*-cells in response to obesity or insulin resistance. On the other hand, the inverse correlation of TMEM27 with BMI in T2D might be explained by a higher metabolic stress in obese people in combination with type 2 diabetes resulting in a progressive loss of *β*-cell function and number. The fact that insulin and BACE2 did not correlate with BMI in our study suggests that TMEM27 might be more strongly up- or downregulated upon the aforementioned compensatory and stress conditions. There is ample evidence from mouse and rat models of obesity and diabetes that *β*-cell mass increases to compensate for the additional insulin demand [13], [14] and that TMEM27 and BACE2 might play a role in regulating *β*-cell mass in *ob/ob* and *db/db* mice and in transgenic mouse models overexpressing TMEM27 or being BACE2 deficient [1], [2]. Albeit still controversial, evidence is accumulating that also in humans *β*-cell number increases under conditions associated with increased insulin demand such as obesity, pregnancy and insulin resistance, and decreases in longstanding T2D [14], [15], [16], [17], [18], [19]. The present finding that TMEM27 expression correlates with BMI in pancreatic tissue specimens of non diabetic patients suggest that higher TMEM27 expression could reflect an increase in *β*-cell number in these individuals. However, whether our approach in determining the *β*-cell area scores and *β*-cell density in human pancreatic islets can be translated into a method to estimate *β*-cell mass *in vivo* in humans remains to be shown.

In conclusion, we established an automated computational pathology approach, which enabled a reliable and objective extraction of pancreatic islet-specific features having as only input histopathological fluorescence images. The simultaneous changes of TMEM27, BACE2, and insulin in the majority of the *β*–cells suggest that these proteins reflect the total number of functional insulin producing *β*–cells. Additionally, *β*–cell subpopulations may be identified which are positive for TMEM27, BACE2 or insulin only. Thus, the cumulative assessment of all three markers may provide further information about the real *β*–cell number per islet and patient.

## Supporting Information

Figure S1 **Area score decision tree and representative stainings.** Decision tree for manual and automated area scoring for islets stained either by immunofluorescence (TMEM27, BACE2, glucagon) or immunohistochemistry (insulin) (**A**). Representative stainings of TMEM27 score (0–3) together with glucagon, BACE2 (score 1–3; no 0 assessed) together with glucagon, and insulin (score 1–3; no 0 assessed) (**B**).
(ZIP)

Figure S2 **Working steps of automated quantification pipeline.** (a) Manual segmentation of the islet. (b) Detection of the cell nuclei based on the dapi channel. (c) Separation of stained area and background. (d) Classification of each pixel into the respective classes of either being positively stained or belonging into background. (e) Classification into *α*- or *β*-cells by counting the total number of stained pixels in a patch around the nuclei for both the 555 and 488 channels. (f) Final computation of the total number of pixels that are classified as stained in the islet (excluding the nuclei areas) normalized with the area of the islet.
(ZIP)

Figure S3 **Variability of islet specific features.** TMEM27 (**A**) and BACE2 (**B**) area score variation quantified with the automated scoring approach. Variation of islet size (**C-D**) and *β*-cell density (**E-F**) assessed either by TMEM27 or BACE2 positive cells.
(ZIP)

Materials and Methods S1 (DOC)
