# Correction: Automated Assessment of β-Cell Area and Density per Islet and Patient Using TMEM27 and BACE2 Immunofluorescence Staining in Human Pancreatic β-Cells

**DOI:** 10.1371/journal.pone.0109467

**Published:** 2014-09-23

**Authors:** The PLOS ONE Staff

The seventh and eighth authors, Holger Moch and Giatgen A. Spinas, should be noted as contributing equally to this work.
