# Supplementary material for: Automated Assessment of β-Cell Area and Density per Islet and Patient Using TMEM27 and BACE2 Immunofluorescence Staining in Human Pancreatic β-Cells
Source: PLoS One. 2014 Jun 6;9(6):e98932. doi: 10.1371/journal.pone.0098932 (PMC4048234; doi:10.1371/journal.pone.0098932)
Supplement: Materials and Methods S1 — (DOC) [file pone.0098932.s004.doc]

**Supplementary Materials and Methods**

**MAIN MATLAB CODE FOR ISLET-SPECIFIC FEATURES ESTIMATION**

% Having a manual islet segmentation (variable islet_mask),

% three staining channels 555 (variable I2), 488 (variable I3)

% and dapi (variable dapi) and the cell nuclei coordinates

% (variable nodes), compute the automated immunofluorescence estimator,

% as welal as other islet-specific features.

% The code is not intended to be distributed and used directly as it

% requires the specification of several data directories, as well as data

% like the coordinates of cell nuclei.

%

% The code is provided to the interested reader in order to understand

% the methods described in the paper.

%

% Xenofon Floros

% ML group ETHZ

% March 2014

%% Clean UP and Load Data

clear all;

close all;

%%%%%%%%%%% SELECT PARAMETERS for analysis %%%%%%%%%%%

staining = {'tmem', 'bace2'};

% Specify the indices of the folders - patients we are interested in

fold_ind = {'03' '04'};

%%%%%%%%%%%%%%%%%%%%%%%%%%%%%%%%%%%%%%%%%%%%%%%%%%%%%

%%

fprintf('------- Batch Feature Extraction launched -------- \n');

for s = 1:length(staining)

% Here we assume that all the folders have the same prefix and a number in

% the end differentiates them.

fold_prefix = ['quant_' staining{s} '_p'];

for f = 1:length(fold_ind) % Loop over the FOLDERS

patient_fold = [fold_prefix fold_ind{f}];

% Find which islet images you have in each folder

indices = find_files_in_folder([dir_original_images patient_fold]);

staining_intens_norm = zeros(1,length(indices));

stained_area_norm = zeros(1,length(indices));

islet_size = zeros(1,length(indices));

bCell_density = zeros(1,length(indices));

for i = 1:length(indices) % Loop over the ISLETS in the folder

fprintf('\n');

fprintf('Extracting staining from patient %6s, islet #%2s ...\n', patient_fold, indices{i});

% Load the DAPI channel

load([dir_segmented_images patient_fold '/' indices{i} '_islet.mat']);

dapi = imread([dir_original_images patient_fold '/' indices{i} '_dapi.tif']);

% Load the cell nuclei coordinates

fileCenters = [dir_nuclei_centers patient_fold '/' indices{i} '_cell_nuclei.dat'];

nodes = csvread(fileCenters);

% Compute DAPI mask for the cell nuclei to exclude later on the

% cell nuclei from the staining estimation.

dapi_intens = double(dapi(:));

cut_thres = mean(dapi_intens) + std(dapi_intens);

dapi_mask = dapi > cut_thres;

dapi_mask(~islet_mask) = 0;

% Compute staining mask

% I is the staining channel

islet_stain = I;

islet_stain(~islet_mask) = 0;

islet_stain(dapi_mask) = 0;

if (strcmp(staining, 'tmem'))

I_channel = I3;

else

I_channel = I2;

end

stain_intens = double(I_channel(:));

% Cluster the staining intensities in 2 groups: staining and

% background.

[indices_clustered, temp] = kmeans(stain_intens,2,...

'distance','sqEuclidean', 'start', [10 ; 90]);

% The adaptive threshold will be the maximum intensity value that

% separates the two groups.

cut_thres = max(stain_intens(indices_clustered==1));

stain_mask = islet_stain > cut_thres;

stain_mask = stain_mask & ~dapi_mask;

stain_amount = I_channel(stain_mask);

stained_area = length(find(stain_mask));

islet_area = length(find(islet_mask));

%%%%% COMPUTE B-CELL DENSITY %%%%%%%%%%%%%%%

% Compute nuclei labels

voting = 0.1;

[nuclei_labeled] = classify_nuclei(I2, I3, nodes, voting);

betaCells_nuclei = nuclei_labeled(nuclei_labeled(:,3)==2,1:2);

ind = sub2ind(size(islet_mask), betaCells_nuclei(:,2), betaCells_nuclei(:,1));

nodes_segmented = false(size(islet_mask));

nodes_segmented(ind) = true;

% Keep only the nodes segmented

nodes_segmented(~islet_mask) = false;

[row, col] = find(nodes_segmented);

betaCells_in_islet = length(row);

%%%%%%%%%%%%%%%%%%%%%%%%%%%%%%%%%%%%%%%%%%%%%%%

% Quantities of interest

staining_intens_norm(i) = sum(stain_amount) / islet_area;

stained_area_norm(i) = stained_area / islet_area;

islet_size(i) = islet_area;

bCell_density(i) = betaCells_in_islet / islet_area;

end % END Loop over the ISLETS in the folder

stainIntens_perPatient{f} = staining_intens_norm;

% THIS IS THE AUTOMATED ESTIMATOR USED IN THE PAPER

stainedArea_perPatient{f} = stained_area_norm;

%%%%%%%%%%%%%%%%%%%%%%%%%%%%%%%%%%%%%%%%%%%%%%%%%

isletSize_perPatient{f} = islet_size;

bCells_perPatient{f} = bCell_density;

end % END of Loop over the FOLDERS-PATIENTS

end % END of Loop over the different stainings specified

**FUNCTION CLASSIFY_NUCLEI()**

function [nodes] = classify_nuclei(I2, I3, m, voting)

% Classify detected nuclei cells into normal, alpha and beta.

% The classification is based on clustering the staining intensities

% into two groups, background vs alpha/beta cells and looking at the

% corresponding channels to make the final decision between alpha and beta

% using a majority voting scheme.

% The first two columns specify the coordinates of the node and the last

% one the label, i.e 1 or 2 for the alpha or beta cells that specify the islet

% and 0 otherwise

% The code is not intended to be distributed and used directly as it

% requires the specification of several data directories, as well as data

% like the coordinates of cell nuclei.

% The code is provided to the interested reader in order to understand

% the methods described in the paper.

%

% Xenofon Floros

% ML group ETHZ

% March 2014

%% Classify Nuclei

nodes = [m zeros(size(m,1),1)];

patchRad = 15;

% Define the percentage of pixels you want to be 1 in each patch in order

% to accept it as 1. 0.5 in majority vote, e.g majority_vote = 0.1;

majority_vote = voting;

alpha_intens = double(I2);

alpha_intens = alpha_intens(:);

beta_intens = double(I3);

beta_intens = beta_intens(:);

% Segment I2 to find possible candidates for alpha cells

% Cluster the histogram in 2 groups

[ind_temp, alpha_centers] = kmeans(alpha_intens,2,...

'distance','sqEuclidean', 'start', [10 ; 90]);

% Find which indices correspond to the alpha cells

alpha_lab = 1;

if (length(ind_temp(ind_temp==1)) > length(ind_temp(ind_temp==2)))

alpha_lab = 2;

end

ind_alpha = ind_temp==alpha_lab;

alpha_mask = zeros(size(I2));

alpha_mask(ind_alpha) = 1;

[MASK_MAX_Y MASK_MAX_X] = size(alpha_mask);

%Segment I3 to find possible candidates for beta cells

% Cluster the histogram in 2 groups

[ind_temp, beta_centers] = kmeans(beta_intens,2,...

'distance','sqEuclidean', 'start', [10 ; 100]);

% Find which indices correspond to the beta cells

beta_lab = 1;

if (length(ind_temp(ind_temp==1)) > length(ind_temp(ind_temp==2)))

beta_lab = 2;

end

ind_beta = ind_temp==beta_lab;

beta_mask = zeros(size(I3));

beta_mask(ind_beta) = 1;

% Make the final classification

for i=1:size(m,1)

x = m(i,1);

y = m(i,2);

if (y-patchRad > 1) y_min = y-patchRad; else y_min = 1; end;

if (y+patchRad < MASK_MAX_Y) y_max = y+patchRad; else y_max = MASK_MAX_Y; end;

if (x-patchRad > 1) x_min = x-patchRad; else x_min = 1; end;

if (x+patchRad < MASK_MAX_X) x_max = x+patchRad; else x_max = MASK_MAX_X; end;

patch2 = alpha_mask(y_min:y_max, x_min:x_max);

patch3 = beta_mask(y_min:y_max, x_min:x_max);

if ( sum(sum(patch2)) > sum(sum(patch3)) )

if ( sum(sum(patch2)) > majority_vote*size(patch2,1)^2 )

nodes(i,3) = 1; % Alpha cells

end

elseif ( sum(sum(patch3)) > majority_vote*size(patch3,1)^2 )

nodes(i,3) = 2; % Beta cells

end

if ( sum(sum(patch3)) > sum(sum(patch2)) )

if ( sum(sum(patch3)) > majority_vote*size(patch3,1)^2 )

nodes(i,3) = 2; % Beta cells

end

elseif ( sum(sum(patch2)) > majority_vote*size(patch2,1)^2 )

nodes(i,3) = 1; % Alpha cells

end

end
